# Trait means predict performance under water limitation better than plasticity for seedlings of Poaceae species on the eastern Tibetan Plateau

**DOI:** 10.1002/ece3.6108

**Published:** 2020-02-12

**Authors:** Honglin Li, Xilai Li, Xiaolong Zhou

**Affiliations:** ^1^ State Key Laboratory of Plateau Ecology and Agriculture Qinghai University Xining China; ^2^ College of Agriculture and Animal Husbandry Qinghai University Xining China; ^3^ Institute of Arid Ecology and Environment Xinjiang University Urumqi China; ^4^ Key Laboratory of Oasis Ecology of the Ministry of Education Xinjiang University Urumqi China

**Keywords:** climate change, functional trait, plasticity, Poaceae, Tibetan Plateau, trait means, water availability

## Abstract

Water availability may be altered by changes in precipitation under global climate change in alpine areas. Trait means and plasticity are important for plants in response to a changing environment. In an examination of alpine plant responses to changed water availability, and for determination of how trait means and plasticity predict the performance (e.g., biomass) of these species, seeds of ten Poaceae species from the eastern Tibetan Plateau were sown and grown in a manipulated environment during a growing season in which rainfall was removed and other climate conditions remained unchanged. Growth and leaf traits of these species were measured. We found significant effects of moderate water stress on the seedling biomass of these species; however, the responses of these species to changed water condition were strongly dependent on species identity. For example, the biomass of some species significantly decreased under moderate drought, whereas that of others were either significantly increased or unaffected. This pattern was also observed for growth and leaf traits. Overall, the alpine Poaceae species showed low plasticity of traits in response to water availability relative to reports from other areas**.** Notably, the results show that trait means were better correlated with the productivity than with the plasticity of traits; thus, we argue that the trait means were better predictors of performance than plasticity for alpine Poaceae species. Poaceae species in alpine areas are important for forage production and for water catchment health worldwide, and these species may face water shortage because of current and future climate change. Understanding the response of alpine Poaceae species to water availability would facilitate our ability to predict the impacts of climate change on the alpine vegetation.

## Graphical abstract

In this study, we found species‐specific responses of alpine Poaceae species to the relative‐drought and well‐watered conditions, and varied plasticity of traits. Our results also showed that trait mean was better correlated with the productivity than the plasticity of traits; thus, we argue that the trait mean was better predictor of performance than plasticity for alpine Poaceae species.

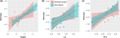

## 1 INTRODUCTION

Understanding plant and soil water relationships is important for management and conservation of terrestrial ecosystems, since the growth of individual plants, and thus the distribution of flora, is determined by soil water conditions (Bernacchi & VanLoocke, 2015; Kramer, 1969). Global climate change is substantially altering the precipitation regimes worldwide (IPCC, 2013; Pfahl, O'Gorman, & Fischer, 2017). Alpine areas, among the most sensitive regions to climate change (Briceño, Harris‐Pascal, Nicotra, Williams, & Ball, 2014; Liu & Chen, 2000; Thompson et al., 2000), might experience a significant change in precipitation (Xu, Gong, & Li, 2008). Therefore, plant species in alpine areas would face great changes in water availability as a consequence of altered precipitation regime (Immerzeel, van Beek, & Bierkens, 2010; Wu, Ren, Wang, Shi, & Warrington, 2013), and in some areas, the situation might become even worse (Xu et al., 2015). So far, little is known about the response of plant growth and functional traits to changed soil water availability in the alpine grassland, especially in the eastern Tibetan Plateau.

Water stress has been considered a cause of variable impacts on plant growth (Bernal et al., 2015; Chaves, Maroco, & Pereira, 2003; Llorens, Penuelas, Estiarte, & Bruna, 2004; del Pozo et al., 2016), and the responses of plant species to water stress may vary among species, as mediated by their functional traits (Körner, 2003; Llorens et al., 2004; Smirnoff, 1993; Violle et al., 2007). Considering a plant species as having a set of morphological and physiological traits which have impacts on the performance of plants (Violle et al., 2007) greatly increases our possibility for examining the differences among plant species in response to environmental variability. For example, study explicitly showed that varied responses of plant species to changes in water availability are associated with variable functional traits (Hernández, Vilagrosa, Pausas, & Bellot, 2010). Thus, examining the responses of plant traits to water availability helps us to predict how plants in the Tibetan Plateau will respond under future changed water regimes.

Most organisms, especially sessile plants, change their phenotype in response to shifts in the environment (referred to as phenotypic plasticity), a strategy plants employ to adapt to habitat change and environmental heterogeneity (Nicotra et al., 2010) and a mechanism that drives adaptive evolution, species coexistence, and ecosystem processes (Turcotte & Levine, 2016; Walker et al., 2019). Furthermore, arguably, the ecological breadth of species may be explained partly by their capacity to show plastic responses to environmental cues (Saldaña, Gianoli, & Lusk, 2005; Sultan, 2001), and these plastic responses are key mechanisms underlying the rapid adjustment to changing environments (Pigliucci, 2001). For example, plasticity of root traits in grassland herbaceous species was important factors in maintaining competitive abilities and resource uptake in response to a changing water supply (Fort, Cruz, & Jouany, 2014; Padilla et al., 2013); moreover, the adaptive effects of plasticity on fitness have also been observed for tree species (Dudley & Schmitt, 1996; Valladares, Wright, Lasso, Kitajima, & Pearcy, 2000b). However, some authors have argued that plasticity of a trait did not necessarily imply provision of a fitness benefit (see van Kleunen & Fischer, 2005), because of costs exist for plasticity, which may exert limitations upon that plasticity (Valladares, Gianoli, & Gómez, 2007). For instance, plasticity may have costs when fitness of a genotype being reduced by a phenotype being expressed through plastic rather than fixed development, since this approach requires the maintenance of sensory abilities and leads to less stable development. (van Kleunen & Fischer, 2005). Alternatively, many studies have shown that the functional traits, rather than plasticity, are good predictors of plant performance in a range of plant species (Poorter & Bongers, 2006; Pywell et al., 2003; Walker et al., 2019) and have suggested that differences in trait values or means were well correlated with differences in average fitness among species (Kraft, Godoy, & Levine, 2015).

Plant growth and evolution in a changed environment lead either to specialization for a given circumstance or to general adaptation to a broad range of environments (Bazzaz, 1996). While phenotypic plasticity is often cited as a characteristic of generalists, trait means (or values) indicate that species specialization applies only to a fraction of the environmental heterogeneity. For example, species with high plasticity have evolved to maximize growth when water is plentiful, whereas species with low plasticity have specialized to adaptations for only water limiting conditions, or only conditions of high‐water availability (Comita & Engelbrecht, 2009). Therefore, a comparison of the relative importance of plasticity and trait means (or values) is needed (Godoy, Valladares, & Castro‐Díez, 2011).

Although a significant correlation has been documented between plasticity and performance (e.g., relative biomass) for plants in an alpine wetland that has experienced fluctuating water conditions during the growing season (Li et al., 2016), other studies conducted in alpine meadows in this area have found significant relationships between the performance (e.g., biomass) and the trait means (Zhou et al., 2017, 2016). Therefore, the role of trait means and plasticity in determining the performance of alpine plant species has shown less consistency, especially for seedlings of Poaceae species, which play particularly important roles in the recruitment and dynamics of grassland communities in the area.

Ten Poaceae species in the eastern Tibetan Plateau were grown under manipulated water conditions, and their growth and leaf traits were investigated to address alpine plant species responses to changing water availability and to evaluate the relative importance of trait means and plasticity in predicting the performance of these species as future changes occur in the water environment. Specifically, the following questions were asked:

1. How do the functional traits of these species respond to different water conditions? How they are influenced by the ecological characteristics of these species?
2. How does the plasticity of the investigated traits differ among the species?
3. Are trait means or plasticity of these species good predictors of the performance (biomass) of these species in response to changing water availability?

## 2 MATERIALS AND METHODS

### 2.1 Study site, species, and seed collection

The study was conducted in a nursery at the Field Station of Lanzhou University in Maqu County. The site is located in the eastern Tibetan Plateau of China (33°59′N, 102°00′E, elevation 3,660 masl). The annual temperature and precipitation (from 1975 to 2010) were 1.2°C and 620 mm, respectively (Niu et al., 2014). The soil texture is classified as a subalpine meadow soil with gravelly, sandy loam and slightly alkaline soils (Gong, 1999). The mean soil nitrogen is 0.556% (Borer et al., 2014). The plant community type is mainly meadow and is affected by degradation (Yu et al., 2012).

The selected species represent a range of Poaceae species common in our study area (Table 1). We chose the ten species that are either common (relative abundance > 1%) or dominant (relative abundance > 10%) across various communities in the region (our field survey data). All species are perennial herbaceous plants except for *Beckmannia syzigachne*, which is common in wet habitats. *Elymus nutans* is the dominant species of the alpine meadow (Chu et al., 2008), and others are also abundant and widely distributed in our study area.

**Table 1:** Species names, habitats, growth types (A, annual; P, perennial), and abbreviations for 10 Poaceae species

Species	Habitat	Growth type	Abbreviation
*Agrostis gigantea*	Meadow	P	AG
*Agrostis perlaxa*	Wetland	P	AP
*Beckmannia syzigachne*	Wetland	A	BS
*Deschampsia caespitosa var. microstachya*	Meadow	P	DC
*Elymus nutans*	Meadow	P	EN
*Festuca ovina*	Wetland	P	FO
*Poa pachyantha*	Meadow	P	PP
*Poa pratensis*	Meadow	P	PPr
*Ptilagrostis dichotoma*	Meadow	P	PD
*Stipa aliena*	Meadow	P	SA

Bulk seed collections for each of the 10 species were collected during a period from late August to mid‐September in 2010. For each species, we chose three to five plants in an area 5 × 5 m (assumed to be same population) in the habitat where they most commonly distributed, and at least 200 seeds were collected from nine to fifty tillers of the cluster. The collected seeds were then stored in a cool dry room until ready for sowing. Seed preparation included extracting seeds from the spike and discarding damaged ones.

### 2.2 Experimental design

In early May 2011, seeds of the ten species were sown into plastic pots (height of 14 cm, diameter of 22 cm at the top and 20 cm at the bottom), and these pots were manipulated to include a plastic‐covered metal shelter erected over the pots to prevent the pots from receiving natural rainfall, with 25 seeds per pot and 12 pots per species (see Table S1 for differences between those inside and outside of the shelter). The pots were filled with soil from a nearby alpine meadow; the soil had been carefully sieved, mixed, and heated to remove the soil seed bank. The pots of each species (*n* = 12) were randomly assigned to two water regimes: well‐watered and exposed to moderate drought. For each treatment, the pots were randomly placed on plastic sheets under the shelter at a minimum distance of 3 cm from each other. The plastic sheets were laid on the ground to prevent the seedlings from accessing ground moisture. The soil water capacity (SWC) was determined by measuring the water‐saturated soil with a portable probe (WET‐2 sensor, Delta‐T Devices Ltd), and the mean soil water capacity was 72.4 ± 2.59 v/v%. The well‐watered treatment was applied at 0.2 L water for each pot, with minimal leakage, and the soil water content remained at approximately 90%–100% of SWC, whereas the treatment exposed to moderate drought was given only 0.1–0.12 L water to keep the soil relatively dry (50%–60% of SWC). The seedlings were watered every third day, and regular measurement of the soil moisture guaranteed the success of these two treatments during the period of the experiment (May–September).

### 2.3 Data collection

The initial harvest (*t*_1_) of seedlings of these species was conducted 1 week after the seed had germinated. One seedling per pot was randomly selected for measuring the initial dry weight (*W*_1_). Then, at least three to five well‐spaced seedlings were kept, and the others were removed from each pot (seedlings germinated later also were removed once they appeared). Since those seedlings left in the pot were well spaced (>5 cm), we assumed competition was minimal. In early September, after more than 2 months of seedling growth, all pots were subjected to final harvest (*t*_2_, the total time during which the seedlings grown were counted from 1 week after they germinated). At this stage, for each pot, we first removed the pot gently and then washed the soil away with running water; finally, three seedlings were selected from each pot for harvest. In total, we obtained 18 seedlings per species per treatment except for *B. syzigachne* (*n* = 15 seedlings). The seedlings were separated into shoot and root, and the biomass of each seedling (*W*_2_) was determined by summing the dry weight of the shoot and root. Meanwhile, one fully expanded leaf was selected from each seedling, the area of the leaf was measured by scanning leaves and analyzing the images with the ImageJ software, and the dry weight of each leaf was weighed. All dry weights were determined by oven‐drying materials at 80°C for 48 hr. Seven traits (See Table 2) commonly used in ecological investigations were identified according to standardized methods (Cornelissen et al., 2003).

**Table 2:** Traits measured in this study (abbreviations of trait, method of measurement (or computation), and level of measurement)

Trait	Abbreviation	Measurement	Measured on
Biomass	Biomass (g)	The weight of oven‐dried organ for each individual, including shoot and root	individual
Shoot height	Height (cm)	Length from base to growing tip	individual
Relative growth rate	RGR (g/d)	(ln*W*_2_ − ln*W*_1_)/(*t*_2_ − *t*_1_)	individual
Root shoot ratio	*R:S*	The dry weight of root/ dry weight of shoot	individual
Leaf area	LA (m^2^)	The area of leaf blades	leaf
Relative leaf water content	RLWC	((Fresh leaf weight − dry leaf weight)/fresh weight of leaf) × 100%	leaf
Specific leaf area	SLA (m^2^/g)	Leaf area per leaf dry mass	leaf
Photosynthetic rate	μmol CO_2_ m^−2^ s^−1^)	The net photosynthesis rate per unit area of leaf	leaf
Transpiration rate	mmol H_2_O m^−2^ s^−1^	The water loss per unit area of leaf	leaf

The photosynthetic rate and transpiration rate (See Table 2 for description) were measured for leaves of seedling from three pots randomly selected for each treatment. For each pot, one fully expanded, newly developed, healthy leaf was selected from two seedlings (in total, six leaves per species per treatment were measured). The measurement was conducted between 1,000 and 1,230 hr during a sunny, clear day (1 day after watering for the dry‐down treatment) in late July with a GFS‐3000 portable photosynthesis system (GFS‐3000; Heinz Walz). The measurements were taken at 1,800 µmol m^−2^ s^−1^, which equates to the average light intensity of sunlight during the measurement (measured by the ambient light sensor of GFS‐3000). The CO_2_ concentration was ∼340 ppm (reflecting the measured ambient concentration on site).

### 2.4 Statistical analysis

The plasticity of the traits in response to different water availability was used for assessing species‐specific plasticity across the treatments. For all traits (except for biomass), we calculated the plasticity index (PI) with the following equation: PI = (maximum trait mean − minimum trait mean)/maximum trait mean (Gratani, Meneghini, Pesoli, & Crescente, 2003; Valladares, Sanchez‐gomez, & Zavala, 2006; Valladares, Wright, et al., 2000b), where the PI ranges from zero (no plasticity) to one (maximum plasticity). The trait means included the means of the 18 plants from each water treatment for each species. Because the maximum value was achieved at low water for some species and at high water for others, the index reflects the absolute value of plasticity rather than the direction of the response.

The effects of the treatments, species identity, and their interaction on the investigated traits were analyzed by two‐way ANOVA, with species and treatment as the fixed factor. The difference of traits between the treatments for each species was compared using a Kruskal–Wallis one‐way ANOVA. Data that violated the ANOVA assumptions of normality and homogeneity of variance were log10‐transformed.

Correlations between biomass and trait means across species were analyzed using a Phylogenetic Generalized Least Squares (PGLS). Phylogenetic tree of our studied species was built based on the Phylomatic (version 3, http://phylodiversity.net/phylomatic/). We also analyzed the correlations between the biomass and the trait plasticity in response to water availability. Data were log10‐transformed. All analysis and plotting were performed with SPSS 16.0 (SPSS) and R (R Core Team, 2019).

## 3 RESULTS

### 3.1 Species‐specific responses of alpine Poaceae species to changes in soil water availability

**Figure 1:**
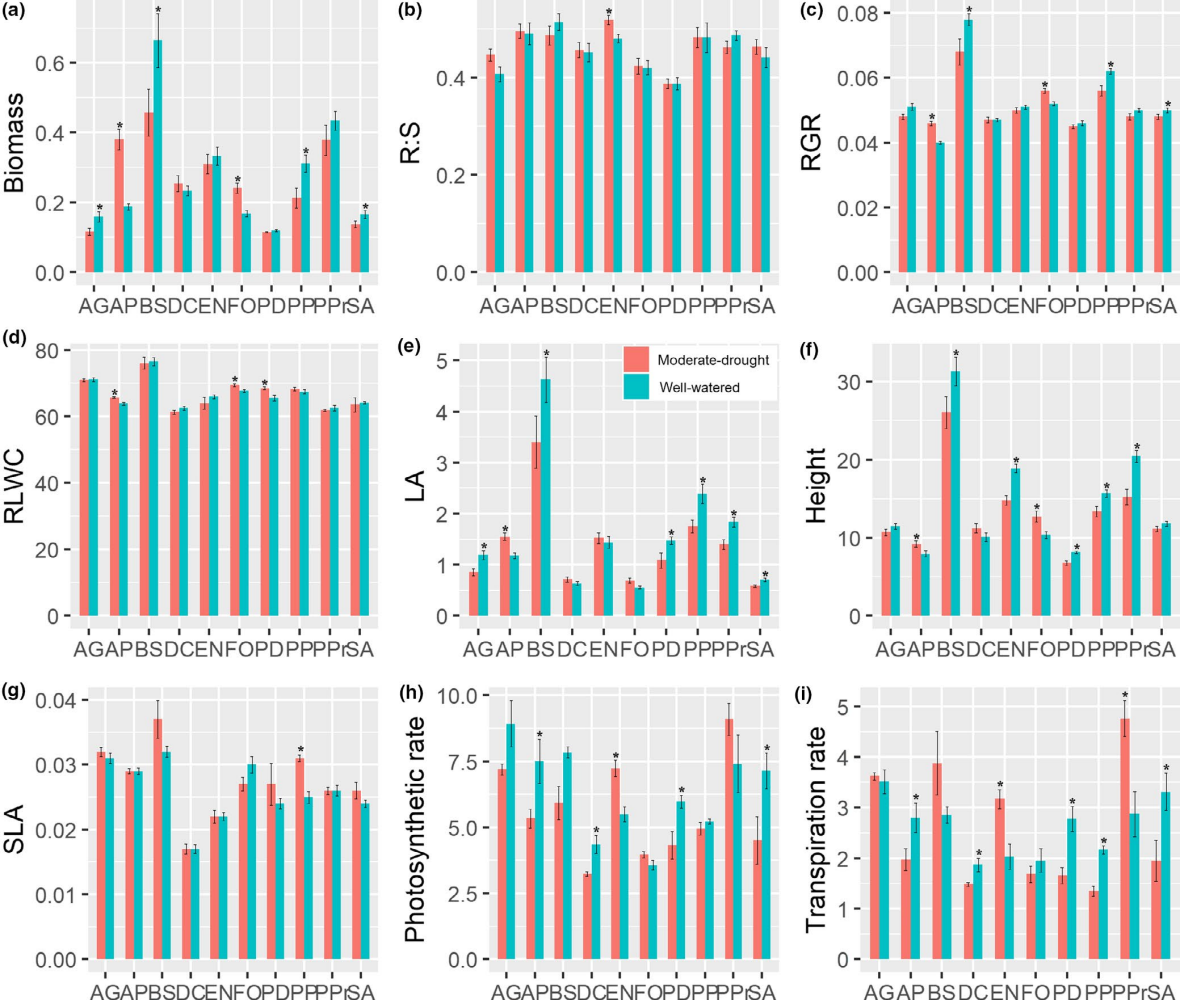
Functional responses of 10 Poaceae species to different conditions of soil water availability. The data are shown as the mean ± *SE*. Asterisks indicate a statistically significant difference between water treatments for the species (*p* < .05). See Table 1 and Table 2 for the abbreviations of species name and traits, respectively

Soil water availability, species identity, and their interaction had significant effects on biomass, including shoot biomass and root biomass (Table 3). Six out of ten species showed significant differences in biomass between the different water treatments (Figure 1a). Further, the response of biomass to water treatment was varied among species; for example, biomass of the four species (AG, BS, PP, and SA, see Table 1 for abbreviations of studied species) was significantly lower under moderate‐drought treatment, whereas that of AP and FO were significantly higher under drought than under well‐watered treatment (Figure 1a). Notably, EN, the dominant Poaceae species in the alpine meadow of the eastern Tibetan Plateau, had relative high biomass under both treatments (Figure 1a).

**Table 3:** Results from analysis of variance of biomass and leaf traits for 10 Poaceae species grown under two different conditions of water availability

Traits	Adjusted *R* ^2^	Treatment		Species		Treatment × Species	
		*F*	*p*	*F*	*p*	*F*	*p*
Biomass	.73	6.84	**.009**	91.20	**<.001**	8.72	**<.001**
Shoot biomass	.73	7.39	**.007**	**<0.001**		9.20	**<.001**
Root biomass	.70	5.10	**.025**	**<0.001**		7.15	**<.001**
RGR	.72	7.69	**.006**	96.96	**<.001**	8.24	**<.001**
*R:S*	.24	0.16	.694	12.41	**<.001**	1.27	.254
Height	.78	20.93	**<.001**	121.27	**<.001**	6.83	**<.001**
SLA	.59	5.32	**.022**	51.71	**<.001**	2.42	**.011**
LA	.81	12.75	**<.001**	153.78	**<.001**	6.23	**<.001**
RLWC	.51	0.3	.587	39.00	**<.001**	1.49	.150
Photosynthetic rate	.62	6.09	**.015**	31.47	**<.001**	6.59	**<.001**
Transpiration rate	.46	4.13	**.044**	13.38	**<.001**	7.71	**<.001**

Note

The model of type III sum of squares was performed at the 0.05 level, with treatment and species as the main effects (degree of freedom for treatment = 1, for species = 9). The adjusted *R* ^2^ is the proportion of total variance explained by the model. Significant values are in bold.

Alongside biomass, other morphological and physiological traits were also significantly affected by species identity, water treatment, and their interaction (Table 3). For example, the relative growth rate (RGR), height, specific leaf area (SLA), leaf area (LA), photosynthetic rate, and transpiration rate were significantly affected by the treatment, species, and their interactions, whereas the root:shoot ratio (*R:S*) and relative leaf water content (RLWC) were only significantly influenced by the species identity (Table 3). Specifically, the *R:S* of all species except for EN were hardly affected by water availability, and EN showed a significantly higher *R:S* under moderate drought than well‐watered (Figure 1b). The RGR of three species (BS, PP, and SA) was significantly lower under moderate drought than well‐watered, whereas the ranking was reversed for AP and FO (Figure 1c). Although all species showed a similar amount of water content in the leaf (>60%), some species (AP, FO and PD) had greater RLWC under moderate drought than under well‐watered conditions (Figure 1d). BS had the largest LA among all species, with significant difference between treatments; AG, PP, PPr, PD, and SA showed a significantly larger LA under the well‐watered treatment than under the treatment with moderate drought, whereas AP showed the opposite ranking (Figure 1e). The seedlings of five species (EN, PP, PPr, BS, and PD) were taller under the well‐watered condition than those grown under conditions of moderate drought, whereas an opposite pattern was observed for AP and FO (Figure 1f). Although most species showed slightly higher SLA under moderate drought than under well‐watered condition, no significant difference was observed for SLA between treatments (except for PP; Figure 1g). Species‐specific responses of photosynthetic rate to changing water conditions were also shown for our studied species; for example, AP, DC, PD, and SA showed significantly higher photosynthetic rate under well‐watered condition than under moderate drought, whereas EN showed the opposite pattern (Figure 1h). Similar patterns were observed in the transpiration rate, with AP, DC, PP, PD, and SA showing significantly higher values and En and PP showing significantly lower values under well‐watered conditions than under the moderate‐drought condition (Figure 1i).

### 3.2 Plasticity

**Figure 2:**
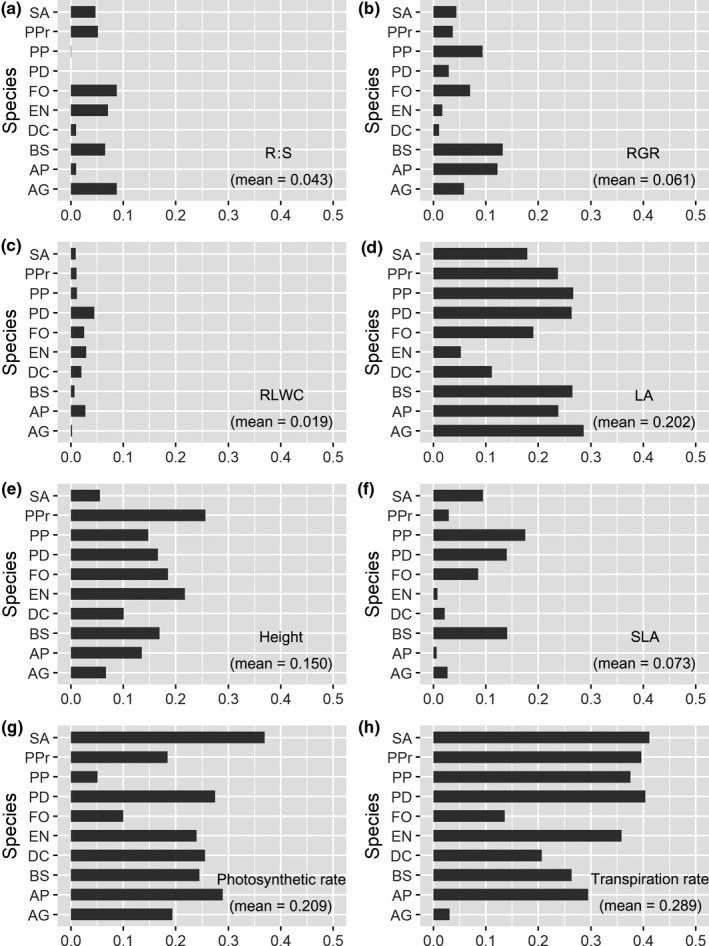
Plasticity index (PI) of growth, leaf, and photosynthetic traits for 10 Poaceae species. See Table 1 and Table 2 for the abbreviations of species name and traits, respectively

The plasticity of the traits in response to water availability varied among the traits and species (Figure 2). Overall, the transpiration rate (mean = 0.289) and photosynthetic rate (mean = 0.202) showed larger plasticity relative to the other traits (e.g., mean value of 0.043 for *R:S* and 0.073 for SLA), whereas the PI of RLWC was the lowest (mean = 0.019). Strong differences also existed among species for plasticity (Figure 2).

### 3.3 Relationships among biomass, trait, and plasticity

**Figure 3:**
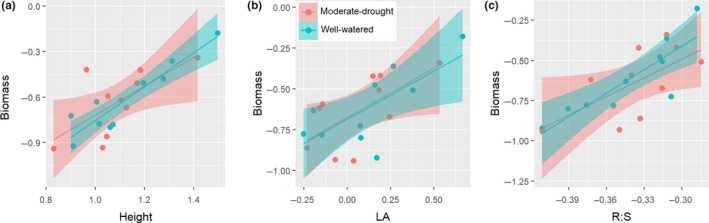
Relationships between biomass and the trait mean of height (a), LA (b), and *R:S* (c) under well‐watered treatment and moderate‐drought treatment. Data were log10 transformed. The shaded area represents the 95% confidence interval of the prediction. Height (well‐watered: *y* = −1.869 + 1.115*x*, *R* ^2^ = .84, *p* < .001; moderate drought: *y* = −1.671 + 0.953*x*, *R* ^2^ = .38, *p* < .05). LA (well‐watered: *y* = −0.685 + 0.590*x*, *R* ^2^ = .45, *p* < .05; moderate drought: *y* = −0.674 + 0.602*x*, *R* ^2^ = .34, *p* < .05). *R:S* (well‐watered: *y* = 1.058 + 4.890*x*, *R* ^2^ = .69, *p* < .001; moderate drought: *y* = −0.668 + 3.865*x*, *R* ^2^ = .33, *p* < .05)

We found significant positive relationships between the biomass and trait means for height, LA, and *R:S* under both well‐watered and moderate‐drought treatments (Figure 3). However, we did not find any significant correlations between the biomass and plasticity for any traits, nor did we observe correlations between the biomass differences and trait plasticity (Table S2).

## 4 DISCUSSION

Using a common garden experiment, we assessed the response of ten common Poaceae species of the eastern Tibetan Plateau to changes in water availability, and we found significant effects of moderate water stress on the plant performance (i.e., biomass). We also revealed species‐specific responses of these species to changes in water availability and variable plasticity for the studied traits among species. In addition, our results suggest that trait mean, rather than plasticity of these Poaceae species were better predictors of their performance in the face of changes in water availability (both low and well water conditions).

### 4.1 Species‐specific variation in response to soil water availability

The substantial interspecific variation of the surveyed species in response to soil water availability confirmed expectations that species would exhibit different responses to the changes in water condition, and this might result from a differential capacity to compete for water (Leyer, 2005; Prechsl, Burri, Gilgen, Kahmen, & Buchmann, 2015). Körner (2003) suggested that alpine species probably have not experienced a shortage in water, and thus, we expected that the alpine species should be more sensitive to a water shortage. The results explicitly suggest that alpine species show a critical sensitivity to water supply during the growing season, and the water supply is projected to change with future climate conditions (Chen et al., 2017; Immerzeel et al., 2010; Wu et al., 2013).

Generally, water stress may reduce plant growth (Bernal et al., 2015; Chaves et al., 2003; Chavoshi, Nourmohamadi, Madani, Hossein, & Mojtaba, 2018; Llorens et al., 2004), decrease the specific leaf area (SLA), and increase the root biomass (e.g., *R:S* ratio) (Benjamin, Nielsen, Vigil, Mikha, & Calderon, 2014; Poorter et al., 2012), plant height, and the leaf area (LA) (Grassein, Till‐Bottraud, & Lavorel, 2010; Nicotra, Hermes, Jones, & Schlichting, 2007). And these changes in plant traits might facilitate function under water stress. In our study, most species tended to have lower biomass, relative growth rate (RGR), height, LA, and photosynthetic rate and higher *R:S* and transpiration rate under moderate‐drought conditions, which was consistent with previous studies (e.g., Bernal et al., 2015; Chavoshi et al., 2018; Nicotra et al., 2007). However, we found similar RLWC across species in our study suggested that species likely have structural modifications to maintain similar RLWC. Unsurprisingly, annual herbaceous plants (BS) achieved higher biomass than perennial ones within a growing season, as well as higher RGR, height, and LA. This greater biomass is needed for annual species to grow rapidly and obtain as many resources as possible in the pre‐reproduction phase (Garnier, 1992).

However, in contrast to our expectation, two species (AP and FO) had significantly higher biomass under moderate drought than under well‐watered condition (Figure 1), and interestingly, the seeds of these two species were collected from a wetland habitat (Table 1), where the mean soil water content was higher than that of the alpine meadow (Li et al., 2018). This result may be explained by the substantial differences in microsite between the hummock and hollow in the wetland (Nungesser, 2003; Shen, Tang, & Washitani, 2006), and this is key mechanism underlying relative high species diversity in alpine wetland. In fact, during the field survey, we found that these two species favor relative dry hummock microhabitats, rather than the moist hollow, and seeds of these two species were also collected from the hummock microhabitats.

Furthermore, we also observed that, for some species (four out of 10), the biomass was not affected by change in soil water availability, especially the dominant species of the alpine meadow (EN). On one hand, the EN maintained relative higher productivity under both treatments by allocating significantly more biomass to the underground (e.g., increasing *R:S* ratio, see Poorter et al., 2012; Benjamin et al., 2014) and by decreasing the height of the shoot under relative‐drought treatment (Figure 1b,f). This strategy was important for stress tolerance so that plant species could maintain their dominance (Smith & Knapp, 2003), and it may stabilize productivity in the alpine meadow in these areas under predicted change in precipitation regime. On the other hand, although the amount of water used by growing plants progressively increased to accommodate the increasing biomass, thus increasing the requirement of water for growth, we did not increase the water supply during the experimental period in this study, and this approach might have contributed to the well‐watered plants having actually experienced low water availability, thereby providing a partial explanation for the lack of differences between the treatments for some species and the need to avoid this approach in future research.

Overall, our results show species‐specific responses of the studied species to changes in soil water. This may suggest that these species might employ different strategies in response to different soil water conditions (Grime, 2006) and indicate the niche differentiation within the alpine ecosystem. In addition, these results imply that the possible future water shortage in our study area may be beneficial for some species (e.g., AP and FO), whereas some other species will suffer from the low water condition (e.g., BS, PP, AG, and SA). Moderate water shortages, however, might not influence the dominant Poaceae species (EN).

### 4.2 Plastic responses to soil water availability

Recently, lots of studies have highlighted the importance of intraspecific trait variation (including phenotypic plasticity and genetic variation among populations) for plants in response to environmental change and for maintaining community stability (Henn et al., 2018; Lajoie & Vellend, 2018; Nicotra et al., 2010; Violle et al., 2012). However, the plasticity of functional traits in response to different water conditions was low in our study relative to studies conducted on other flora (Sadras & Trentacoste, 2011), and no significant correlation was observed between the plasticity and the biomass for all species. These results confirmed the other studies that have shown neutral or maladaptive plasticity of drought tolerance traits in response to water limitation in harsh environments, such as desert and arid ecosystems (Donovan, Dudley, Rosenthal, & Ludwig, 2007; Pohlman, Nicotra, & Murray, 2005). They might also reflect the high‐elevation species are less plastic than the low‐elevation plants because of the canalization traits (Nicotra et al., 2015). Since phenotypic plasticity is not always adaptive and often is associated with costs (Valladares et al., 2007; van Kleunen & Fischer, 2005), for those seedlings growing in the alpine area, which are weakened by and sensitive to environmental changes, less plasticity may be a conservative strategy to avoid energy costs and extra risks. In fact, a recent study reported no evidence of adaptive plasticity for seedlings in a grassland (Harrison & Laforgia, 2019). Furthermore, in consideration of the harsh environmental conditions in the alpine area, particularly the risk of freezing damage (e.g., Rixen, Dawes, Wipf, & Hagedorn, 2012; Wheeler et al., 2014), the conservative resource‐use strategy with low plasticity might be more favored in alpine species under constant selection pressure (Valladares, Wright, et al., 2000b).

We observed that the PI of photosynthetic characteristics was higher than that of the morphological and growth traits in our study, indicating that the leaf physiological traits such as photosynthetic rate and transpiration rate were more flexible in response to environmental changes due to their relatively low cost (Li et al., 2016), as well as because they are not fixed after development. More variation was also observed in the productivity of individuals than in the plasticity for functional traits, and this finding was consistent with previous studies on plasticity of some subalpine grassland species (Grassein et al., 2010), where larger plasticity was observed at the individual level than at the leaf level and suggests this plasticity might depend on the resource‐use strategy (Grime & Mackey, 2002; Valladares, Balaguer, Martinez‐Ferri, Perez‐Corona, & Manrique, 2002). Meanwhile, when single species are examined, the dominant species EN, which achieved relatively higher biomass under both treatments, showed higher plasticity for *R:S* and *H* in response to the water availability. This showed that for some species plasticity may adaptive and can increase its fitness (Nicotra et al., 2010), implying that the plasticity of some key traits for some species might be important in response to water availability and may need more attention in future studies.

### 4.3 Attribution of trait means to productivity

Many studies have reported that the functional traits of a plant species can influence the species survival, growth, and reproduction and are good predictors of the plant performance, from individuals to the ecosystem (Ackerly, 2003; Chapin, 2003; Diaz et al., 2004; Lavorel & Garnier, 2002; Poorter et al., 2008; Valladares, Martinez‐Ferri, Balaguer, Perez‐Corona, & Manrique, 2000a; Wright et al., 2004). Our results confirm this and show that the biomass of the surveyed species was significantly correlated with means of some key trait (e.g., *R:S*, Height, and LA), rather than with the plasticity (Figure 3), suggesting that trait means were better than plasticity for predicting the performance of Poaceae species in response to different water availability in the eastern Tibetan Plateau. Across the treatment, the means of *R:S*, *H* and LA were significantly positively correlated to the biomass, indicating that taller species with larger leaves and root biomass could obtain more resources and thus greater biomass. This pattern suggests that competition both belowground and aboveground was important for species to achieve better performance in our study area (Li, Wen, Hu, & Du, 2011). The increased root biomass under water stress would facilitate the uptake of the water and nutrients, whereas a tall plant with large leaves would more easily intercept light, therefore showing better growth and being more competitive.

The seedling of alpine species is a crucial stage for species to recruit new individuals. Because this stage is vulnerable and sensitive to disturbances, in harsh early spring environments, as they emerge and grow, alpine seedlings can reasonably be expected to maintain a conservative strategy to avoid the extra cost of plasticity (van Kleunen & Fischer, 2005). Moreover, the linkage between biomass and trait means might also indicate the local adaptation of these species.

## 5 CONCLUSION

In this paper, we elucidated the responses of Poaceae species to changes in water availability on the eastern Tibetan Plateau and evaluated the adaptive value of the functional traits and its plasticity. Overall, significant effects were observed of moderate water stress on the growth of the Poaceae species, but the responses of these species to changes in water condition differed strongly between species, indicating that the response of the alpine grassland to water shortage will depend greatly upon the species composition. Perhaps due to the harsh environment, the plasticity of these species was relatively low, and no significant relationship existed between the plasticity and the productivity, whereas the relationships between the productivity and the trait means were significant, suggesting that the trait means were better predictors for the performance of these species than plasticity. Our study will facilitate the understanding of alpine plant species in response to changed soil water conditions in a time of significant climate change, which might change the precipitation regime worldwide. Additionally, studies are needed to examine the responses of plants to changes in different environmental factors (such as temperature, nutrition and their interactions) at a different level (from individual to ecosystem) in this area.

## CONFLICT OF INTEREST

No conflict of interest exists in the submission of this manuscript.

## AUTHOR CONTRIBUTIONS

H.L. conceived and designed the experiments. H.L. and X.Z. performed the experiments and collected the data. H.L. analyzed the data. H.L., X.L., and X.Z. contributed to the writing of the manuscript.

## Data Availability

Data associated with this article can be found in the Dryad Digital Repository at https://doi.org/10.5061/dryad.cz8w9gj0j.

## References

[ece36108-bib-0001] Ackerly, D. D. (2003). Community assembly, niche conservatism, and adaptive evolution in changing environments. International Journal of Plant Sciences, 164, 165–184. 10.1086/368401

[ece36108-bib-0002] Bazzaz, F. A. (1996). Plants in changing environments: Linking physiological, population, and community ecology. Cambridge, UK: Cambridge University Press.

[ece36108-bib-0003] Benjamin, J. G. , Nielsen, D. C. , Vigil, M. F. , Mikha, M. M. , & Calderon, F. (2014). Water deficit stress effects on corn (*Zea mays* L.) root: Shoot ratio. Open Journal of Soil Science, 4, 45094.

[ece36108-bib-0004] Bernacchi, C. J. , & VanLoocke, A. (2015). Terrestrial ecosystems in a changing environment: A dominant role for water. Annual Review of Plant Biology, 66, 599–622. 10.1146/annurev-arplant-043014-114834 25621516

[ece36108-bib-0005] Bernal, M. , Verdaguer, D. , Badosa, J. , Abadía, A. , Llusià, J. , Peñuelas, J. , … Llorens, L. (2015). Effects of enhanced UV radiation and water availability on performance, biomass production and photoprotective mechanisms of *Laurus nobilis* seedlings. Environmental and Experimental Botany, 109, 264–275. 10.1016/j.envexpbot.2014.06.016

[ece36108-bib-0006] Borer, E. T. , Seabloom, E. W. , Gruner, D. S. , Harpole, W. S. , Hillebrand, H. , Lind, E. M. , … Yang, L. H. (2014). Herbivores and nutrients control grassland plant diversity via light limitation. Nature, 508, 517–520. 10.1038/nature13144 24670649

[ece36108-bib-0007] Briceño, V. F. , Harris‐Pascal, D. , Nicotra, A. B. , Williams, E. , & Ball, M. C. (2014). Variation in snow cover drives differences in frost resistance in seedlings of the alpine herb *Aciphylla glacialis* . Environmental and Experimental Botany, 106, 174–181. 10.1016/j.envexpbot.2014.02.011

[ece36108-bib-0008] Chapin, F. S. (2003). Effects of plant traits on ecosystem and regional processes: A conceptual framework for predicting the consequences of global change. Annals of Botany, 91, 455–463. 10.1093/aob/mcg041 12588725 PMC4241063

[ece36108-bib-0009] Chaves, M. M. , Maroco, J. P. , & Pereira, J. S. (2003). Understanding plant responses to drought‐from genes to the whole plant. Functional Plant Biology, 30, 239–264. 10.1071/FP02076 32689007

[ece36108-bib-0010] Chavoshi, S. , Nourmohamadi, G. , Madani, H. , Hossein, H. S. , & Mojtaba, A. (2018). The effects of biofertilizers on physiological traits and biomass accumulation of red beans (*Phaseolus vulgaris* cv. Goli) under water stress. Plant Physiology, 8(4), 2555–2562.

[ece36108-bib-0011] Chen, X. , Wang, G. , Zhang, T. , Mao, T. , Wei, D. A. , Song, C. , … Huang, K. (2017). Effects of warming and nitrogen fertilization on GHG flux in an alpine swamp meadow of a permafrost region. Science of the Total Environment, 601, 1389–1399. 10.1016/j.scitotenv.2017.06.028 28605857

[ece36108-bib-0012] Chu, C. J. , Maestre, F. T. , Xiao, S. , Weiner, J. , Wang, Y. S. , Duan, Z. H. , & Wang, G. (2008). Balance between facilitation and resource competition determines biomass–density relationships in plant populations. Ecology Letters, 11, 1189–1197. 10.1111/j.1461-0248.2008.01228.x 18684118

[ece36108-bib-0013] Comita, L. S. , & Engelbrecht, B. M. (2009). Seasonal and spatial variation in water availability drive habitat associations in a tropical forest. Ecology, 90, 2755–2765. 10.1890/08-1482.1 19886485

[ece36108-bib-0014] Cornelissen, J. H. C. , Lavorel, S. , Garnier, E. , Díaz, S. , Buchmann, N. , Gurvich, D. E. , … Poorter, H. (2003). A handbook of protocols for standardised and easy measurement of plant functional traits worldwide. Australian Journal of Botany, 51, 335–380. 10.1071/BT02124

[ece36108-bib-0016] del Pozo, A. , Yáñez, A. , Matus, I. A. , Tapia, G. , Castillo, D. , Sanchez‐Jardón, L. , & Araus, J. L. (2016). Physiological traits associated with wheat yield potential and performance under water‐stress in a Mediterranean environment. Frontiers in Plant Science, 7, 987. 27458470 10.3389/fpls.2016.00987 PMC4936474

[ece36108-bib-0017] Diaz, S. , Hodgson, J. G. , Thompson, K. , Cabido, M. , Cornelissen, J. , Jalili, A. , … Zak, M. R. (2004). The plant traits that drive ecosystems: Evidence from three continents. Journal of Vegetation Science, 15, 295–304. 10.1111/j.1654-1103.2004.tb02266.x

[ece36108-bib-0018] Donovan, L. A. , Dudley, S. A. , Rosenthal, D. M. , & Ludwig, F. (2007). Phenotypic selection on leaf water use efficiency and related ecophysiological traits for natural populations of desert sunflowers. Oecologia, 152, 13–25. 10.1007/s00442-006-0627-5 17165094

[ece36108-bib-0019] Dudley, S. A. , & Schmitt, J. (1996). Testing the adaptive plasticity hypothesis: Density‐dependent selection on manipulated stem length in Impatiens capensis. American Naturalist, 147, 445–465. 10.1086/285860

[ece36108-bib-0021] Fort, F. , Cruz, P. , & Jouany, C. (2014). Hierarchy of root functional trait values and plasticity drive early‐stage competition for water and phosphorus among grasses. Functional Ecology, 28, 1030–1040. 10.1111/1365-2435.12217

[ece36108-bib-0022] Garnier, E. (1992). Growth analysis of congeneric annual and perennial grass species. Journal of Ecology, 80, 665–675. 10.2307/2260858

[ece36108-bib-0023] Godoy, O. , Valladares, F. , & Castro‐Díez, P. (2011). Multispecies comparison reveals that invasive and native plants differ in their traits but not in their plasticity. Functional Ecology, 25(6), 1248–1259. 10.1111/j.1365-2435.2011.01886.x

[ece36108-bib-0024] Gong, Z. T. (1999). Chinese soil taxonomy: Theories methods and applications. Beijing, China: Science Press.

[ece36108-bib-0025] Grassein, F. , Till‐Bottraud, I. , & Lavorel, S. (2010). Plant resource‐use strategies: The importance of phenotypic plasticity in response to a productivity gradient for two subalpine species. Annals of Botany, 106, 637–645. 10.1093/aob/mcq154 20682576 PMC2944977

[ece36108-bib-0026] Gratani, L. , Meneghini, M. , Pesoli, P. , & Crescente, M. F. (2003). Structural and functional plasticity of *Quercus ilex* seedlings of different provenances in Italy. Trees, 17, 515–521. 10.1007/s00468-003-0269-8

[ece36108-bib-0027] Grime, J. P. (2006). Plant strategies, vegetation processes, and ecosystem properties. New York, NY: John Wiley & Sons.

[ece36108-bib-0029] Grime, J. P. , & Mackey, J. M. L. (2002). The role of plasticity in resource capture by plants. Evolutionary Ecology, 16, 299–307. 10.1023/A:1019640813676

[ece36108-bib-0032] Harrison, S. , & LaForgia, M. (2019). Seedling traits predict drought‐induced mortality linked to diversity loss. Proceedings of the National Academy of Sciences of the United States of America, 116(12), 5576–5581. 10.1073/pnas.1818543116 30833396 PMC6431227

[ece36108-bib-0033] Henn, J. J. , Buzzard, V. , Enquist, B. J. , Halbritter, A. H. , Klanderud, K. , Maitner, B. S. , … Vandvik, V. (2018). Intraspecific trait variation and phenotypic plasticity mediate alpine plant species response to climate change. Frontiers in Plant Science, 9, 1548 10.3389/fpls.2018.01548 30483276 PMC6243391

[ece36108-bib-0034] Hernández, E. I. , Vilagrosa, A. , Pausas, J. G. , & Bellot, J. (2010). Morphological traits and water use strategies in seedlings of Mediterranean coexisting species. Plant Ecology, 207, 233–244. 10.1007/s11258-009-9668-2

[ece36108-bib-0035] Immerzeel, W. W. , Van Beek, L. P. , & Bierkens, M. F. (2010). Climate change will affect the Asian water towers. Science, 328, 1382–1385. 10.1126/science.1183188 20538947

[ece36108-bib-0036] IPCC (2013). Climate change 2013: The physical science basis. Contribution of working group I to the fifth assessment report of the intergovernmental panel on climate change. London, UK: Cambridge University Press.

[ece36108-bib-0037] Körner, C. H. (2003). Alpine plant life: Functional plant ecology of high mountain ecosystems. Berlin, Germany: Springer.

[ece36108-bib-0038] Kraft, N. J. , Godoy, O. , & Levine, J. M. (2015). Plant functional traits and the multidimensional nature of species coexistence. Proceedings of the National Academy of Sciences of the United States of America, 112, 797–802. 10.1073/pnas.1413650112 25561561 PMC4311865

[ece36108-bib-0039] Kramer, P. J. (1969). Plant and soil water relationships: A modern synthesis. New York, NY: McGraw‐Hill Book Company.

[ece36108-bib-0040] Lajoie, G. , & Vellend, M. (2018). Characterizing the contribution of plasticity and genetic differentiation to community‐level trait responses to environmental change. Ecology and Evolution, 8(8), 3895–3907. 10.1002/ece3.3947 29721266 PMC5916269

[ece36108-bib-0041] Lavorel, S. , & Garnier, E. (2002). Predicting changes in community composition and ecosystem functioning from plant traits: Revisiting the Holy Grail. Functional Ecology, 16, 545–556. 10.1046/j.1365-2435.2002.00664.x

[ece36108-bib-0042] Leyer, I. (2005). Predicting plant species' responses to river regulation: The role of water level fluctuations. Journal of Applied Ecology, 42, 239–250. 10.1111/j.1365-2664.2005.01009.x

[ece36108-bib-0043] Li, H. , Yu, K. , Ratajczak, Z. , Nippert, J. B. , Tondrob, D. , Xu, D. , … Du, G. (2016). When variability outperforms the mean: Trait plasticity predicts plant cover and biomass in an alpine wetland. Plant and Soil, 407(1–2), 401–415. 10.1007/s11104-016-2898-x

[ece36108-bib-0044] Li, H. , Yu, K. , Xu, D. , Li, W. , Tondrob, D. , & Du, G. (2018). Structural, compositional and trait differences between the mature and the swamp meadow communities. Journal of Plant Ecology, 11(1), 158–167. 10.1093/jpe/rtw132

[ece36108-bib-0045] Li, W. , Wen, S. , Hu, W. , & Du, G. (2011). Root‐shoot competition interactions cause diversity loss after fertilization: A field experiment in an alpine meadow on the Tibetan Plateau. Journal of Plant Ecology, 4, 138–146. 10.1093/jpe/rtq031

[ece36108-bib-0046] Liu, X. , & Chen, B. (2000). Climatic warming in the Tibetan Plateau during recent decades. International Journal of Climatology, 20, 1729–1742. 10.1002/1097-0088(20001130)20:14<1729:AID-JOC556>3.0.CO;2-Y

[ece36108-bib-0047] Llorens, L. , Penuelas, J. , Estiarte, M. , & Bruna, P. (2004). Contrasting growth changes in two dominant species of a Mediterranean shrubland submitted to experimental drought and warming. Annals of Botany, 94, 843–853. 10.1093/aob/mch211 15466877 PMC4242278

[ece36108-bib-0048] Nicotra, A. B. , Atkin, O. K. , Bonser, S. P. , Davidson, A. M. , Finnegan, E. J. , Mathesius, U. , … van Kleunen, M. (2010). Plant phenotypic plasticity in a changing climate. Trends in Plant Science, 15, 684–692. 10.1016/j.tplants.2010.09.008 20970368

[ece36108-bib-0049] Nicotra, A. B. , Hermes, J. P. , Jones, C. S. , & Schlichting, C. D. (2007). Geographic variation and plasticity to water and nutrients in *Pelargonium australe* . New Phytologist, 176, 136–149. 17803645 10.1111/j.1469-8137.2007.02157.x

[ece36108-bib-0050] Nicotra, A. B. , Segal, D. L. , Hoyle, G. L. , Schrey, A. W. , Verhoeven, K. J. , & Richards, C. L. (2015). Adaptive plasticity and epigenetic variation in response to warming in an Alpine plant. Ecology and Evolution, 5, 634–647. 10.1002/ece3.1329 25691987 PMC4328768

[ece36108-bib-0051] Niu, K. , Choler, P. , de Bello, F. , Mirotchnick, N. , Du, G. , & Sun, S. (2014). Fertilization decreases species diversity but increases functional diversity: A three‐year experiment in a Tibetan alpine meadow. Agriculture, Ecosystems & Environment, 182, 106–112. 10.1016/j.agee.2013.07.015

[ece36108-bib-0052] Nungesser, M. K. (2003). Modelling microtopography in boreal peatlands: Hummocks and hollows. Ecological Modelling, 165, 175–207. 10.1016/S0304-3800(03)00067-X

[ece36108-bib-0053] Padilla, F. M. , Aarts, B. H. , Roijendijk, Y. O. , de Caluwe, H. , Mommer, L. , Visser, E. J. , & de Kroon, H. (2013). Root plasticity maintains growth of temperate grassland species under pulsed water supply. Plant and Soil, 369, 377–386. 10.1007/s11104-012-1584-x

[ece36108-bib-0055] Pfahl, S. , O'Gorman, P. A. , & Fischer, E. M. (2017). Understanding the regional pattern of projected future changes in extreme precipitation. Nature Climate Change, 7(6), 423 10.1038/nclimate3287

[ece36108-bib-0056] Pigliucci, M. (2001). Phenotypic plasticity: Beyond nature and nurture. Baltimore, MD: Johns Hopkins University Press.

[ece36108-bib-0057] Pohlman, C. L. , Nicotra, A. B. , & Murray, B. R. (2005). Geographic range size, seedling ecophysiology and phenotypic plasticity in Australian Acacia species. Journal of Biogeography, 32, 341–351. 10.1111/j.1365-2699.2004.01181.x

[ece36108-bib-0058] Poorter, H. , Niklas, K. J. , Reich, P. B. , Oleksyn, J. , Poot, P. , & Mommer, L. (2012). Biomass allocation to leaves, stems and roots: Meta‐analyses of interspecific variation and environmental control. New Phytologist, 193, 30–50. 10.1111/j.1469-8137.2011.03952.x 22085245

[ece36108-bib-0059] Poorter, L. , & Bongers, F. (2006). Leaf traits are good predictors of plant performance across 53 rain forest species. Ecology, 87, 1733–1743. 10.1890/0012-9658(2006)87[1733:LTAGPO]2.0.CO;2 16922323

[ece36108-bib-0060] Poorter, L. , Wright, S. J. , Paz, H. , Ackerly, D. D. , Condit, R. , Ibarra‐Manríquez, G. , … Wright, I. J. (2008). Are functional traits good predictors of demographic rates? Evidence from five Neotropical forests. Ecology, 89, 1908–1920. 10.1890/07-0207.1 18705377

[ece36108-bib-0061] Prechsl, U. E. , Burri, S. , Gilgen, A. K. , Kahmen, A. , & Buchmann, N. (2015). No shift to a deeper water uptake depth in response to summer drought of two lowland and sub‐alpine C3‐grasslands in Switzerland. Oecologia, 177, 97–111. 10.1007/s00442-014-3092-6 25273953

[ece36108-bib-0062] Pywell, R. F. , Bullock, J. M. , Roy, D. B. , Warman, L. I. Z. , Walker, K. J. , & Rothery, P. (2003). Plant traits as predictors of performance in ecological restoration. Journal of Applied Ecology, 40, 65–77. 10.1046/j.1365-2664.2003.00762.x

[ece36108-bib-0063] R Core Team (2019). R: A language and environment for statistical computing. Vienna, Austria: R Foundation for Statistical Computing Retrieved from https://www.R-project.org/

[ece36108-bib-0064] Rixen, C. , Dawes, M. A. , Wipf, S. , & Hagedorn, F. (2012). Evidence of enhanced freezing damage in treeline plants during six years of CO2 enrichment and soil warming. Oikos, 121, 1532–1543. 10.1111/j.1600-0706.2011.20031.x

[ece36108-bib-0065] Sadras, V. O. , & Trentacoste, E. R. (2011). Phenotypic plasticity of stem water potential correlates with crop load in horticultural trees. Tree Physiology, 31(5), 494–499. 10.1093/treephys/tpr043 21636690

[ece36108-bib-0066] Saldaña, A. , Gianoli, E. , & Lusk, C. H. (2005). Ecophysiological responses to light availability in three *Blechnum* species (Pteridophyta, Blechnaceae) of different ecological breadth. Oecologia, 145, 251–256. 10.1007/s00442-005-0116-2 16025357

[ece36108-bib-0067] Shen, H. , Tang, Y. , & Washitani, I. (2006). Morphological plasticity of *Primula nutans* to hummock‐and‐hollow microsites in an alpine wetland. Journal of Plant Research, 119, 257–264. 10.1007/s10265-006-0269-z 16570124

[ece36108-bib-0068] Smirnoff, N. (1993). Tansley Review No. 52. The role of active oxygen in the response of plants to water deficit and desiccation. New Phytologist, 125, 27–58. 10.1111/j.1469-8137.1993.tb03863.x 33874604

[ece36108-bib-0070] Smith, M. D. , & Knapp, A. K. (2003). Dominant species maintain ecosystem function with non‐random species loss. Ecology Letters, 6, 509–517. 10.1046/j.1461-0248.2003.00454.x

[ece36108-bib-0071] Sultan, S. E. (2001). Phenotypic plasticity for fitness components in *Polygonum* species of contrasting ecological breadth. Ecology, 82, 328–343.

[ece36108-bib-0072] Thompson, L. G. , Yao, T. , Mosley‐Thompson, E. , Davis, M. E. , Henderson, K. A. , & Lin, P. N. (2000). A high‐resolution millennial record of the South Asian monsoon from Himalayan ice cores. Science, 289, 1916–1919. 10.1126/science.289.5486.1916 10988068

[ece36108-bib-0073] Turcotte, M. M. , & Levine, J. M. (2016). Phenotypic plasticity and species coexistence. Trends in Ecology & Evolution, 31(10), 803–813. 27527257 10.1016/j.tree.2016.07.013

[ece36108-bib-0074] Valladares, F. , Balaguer, L. , Martinez‐Ferri, E. , Perez‐Corona, E. , & Manrique, E. (2002). Plasticity, instability and canalization: Is the phenotypic variation in seedlings of sclerophyll oaks consistent with the environmental unpredictability of Mediterranean ecosystems? New Phytologist, 156, 457–467. 10.1046/j.1469-8137.2002.00525.x 33873566

[ece36108-bib-0075] Valladares, F. , Gianoli, E. , & Gómez, J. M. (2007). Ecological limits to plant phenotypic plasticity. New Phytologist, 176, 749–763. 10.1111/j.1469-8137.2007.02275.x 17997761

[ece36108-bib-0076] Valladares, F. , Martinez‐Ferri, E. , Balaguer, L. , Perez‐Corona, E. , & Manrique, E. (2000a). Low leaf‐level response to light and nutrients in Mediterranean evergreen oaks: A conservative resource‐use strategy? New Phytologist, 148, 79–91. 10.1046/j.1469-8137.2000.00737.x 33863045

[ece36108-bib-0077] Valladares, F. , Sanchez‐gomez, D. , & Zavala, M. A. (2006). Quantitative estimation of phenotypic plasticity: Bridging the gap between the evolutionary concept and its ecological applications. Journal of Ecology, 94, 1103–1116. 10.1111/j.1365-2745.2006.01176.x

[ece36108-bib-0078] Valladares, F. , Wright, S. J. , Lasso, E. , Kitajima, K. , & Pearcy, R. W. (2000b). Plastic phenotypic response to light of 16 congeneric shrubs from a Panamanian rainforest. Ecology, 81, 1925–1936. 10.1890/0012-9658(2000)081[1925:PPRTLO]2.0.CO;2

[ece36108-bib-0079] Van Kleunen, M. , & Fischer, M. (2005). Constraints on the evolution of adaptive phenotypic plasticity in plants. New Phytologist, 166, 49–60. 10.1111/j.1469-8137.2004.01296.x 15760350

[ece36108-bib-0080] Violle, C. , Enquist, B. J. , McGill, B. J. , Jiang, L. , Albert, C. H. , Hulshof, C. , … Messier, J. (2012). The return of the variance: Intraspecific variability in community ecology. Trends in Ecology & Evolution, 27(4), 244–252. 10.1016/j.tree.2011.11.014 22244797

[ece36108-bib-0081] Violle, C. , Navas, M. L. , Vile, D. , Kazakou, E. , Fortunel, C. , Hummel, I. , & Garnier, E. (2007). Let the concept of trait be functional!. Oikos, 116(5), 882–892. 10.1111/j.0030-1299.2007.15559.x

[ece36108-bib-0082] Walker, T. W. N. , Weckwerth, W. , Bragazza, L. , Fragner, L. , Forde, B. G. , Ostle, N. J. , … Bardgett, R. D. (2019). Plastic and genetic responses of a common sedge to warming have contrasting effects on carbon cycle processes. Ecology Letters, 22, 159–169. 10.1111/ele.13178 30556313 PMC6334510

[ece36108-bib-0083] Wheeler, J. A. , Hoch, G. , Cortés, A. J. , Sedlacek, J. , Wipf, S. , & Rixen, C. (2014). Increased spring freezing vulnerability for alpine shrubs under early snowmelt. Ecologia, 75, 219–229. 10.1007/s00442-013-2872-8 24435708

[ece36108-bib-0084] Wright, I. J. , Reich, P. B. , Westoby, M. , Ackerly, D. D. , Baruch, Z. , Bongers, F. , … Villar, R. (2004). The worldwide leaf economics spectrum. Nature, 428, 821–827. 10.1038/nature02403 15103368

[ece36108-bib-0085] Wu, G. L. , Ren, G. H. , Wang, D. , Shi, Z. H. , & Warrington, D. (2013). Above‐and below‐ground response to soil water change in an alpine wetland ecosystem on the Qinghai‐Tibetan Plateau, China. Journal of Hydrology, 476, 120–127. 10.1016/j.jhydrol.2012.10.031

[ece36108-bib-0086] Xu, D. , Li, H. , Fang, X. , Li, J. , Bu, H. , Zhang, W. , … Si, X. (2015). Responses of plant community composition and eco‐physiological characteristics of dominant species to different soil hydrologic regimes in alpine marsh wetlands on Qinghai‐Tibetan Plateau, China. Wetlands, 35, 381–390. 10.1007/s13157-015-0627-5

[ece36108-bib-0087] Xu, Z. X. , Gong, T. L. , & Li, J. Y. (2008). Decadal trend of climate in the Tibetan Plateau‐regional temperature and precipitation. Hydrological Processes, 22, 3056–3065. 10.1002/hyp.6892

[ece36108-bib-0088] Yu, K. , Pypker, T. G. , Keim, R. F. , Chen, N. , Yang, Y. , Guo, S. , … Wang, G. (2012). Canopy rainfall storage capacity as affected by sub‐alpine grassland degradation in the Qinghai‐Tibetan Plateau, China. Hydrological Processes, 26, 3114–3123. 10.1002/hyp.8377

[ece36108-bib-0089] Zhou, X. , Guo, Z. , Zhang, P. , Li, H. , Chu, C. , Li, X. , & Du, G. (2017). Different categories of biodiversity explain productivity variation after fertilization in a Tibetan alpine meadow community. Ecology and Evolution, 7(10), 3464–3474. 10.1002/ece3.2723 28515882 PMC5433997

[ece36108-bib-0090] Zhou, X. , Wang, Y. , Zhang, P. , Guo, Z. , Chu, C. , & Du, G. (2016). The effects of fertilization on the traitabundance relationships in a Tibetan alpine meadow community. Journal of Plant Ecology, 9(2), 144–152. 10.1093/jpe/rtv043

