# Supplementary material for: Trait means predict performance under water limitation better than plasticity for seedlings of Poaceae species on the eastern Tibetan Plateau
Source: Ecol Evol. 2020 Feb 12;10(6):2944–55. doi: 10.1002/ece3.6108 (PMC7083665; doi:10.1002/ece3.6108)
Supplement: Supplementary file 1 [file ECE3-10-2944-s001.docx]

**Appendix Table 1** Mean (± SD) of photosynthetic active radiation (PAR), temperature (T), relative humidity (RH), concentration of carbon dioxide (CO_2_) inside and outside of the shelter, measured during midday, while sunny, from 10 to 20 July.

| Environmental factors | Inside | Outside |
| --- | --- | --- |
| PAR (μmol m^−2^ s^−1^) | 1798.64 ± 22.50 | 1848.96 ± 20.84 |
| T (°C) | 23.5 ± 1.0 | 22.8 ± 0.6 |
| RH (%) | 62.49 ± 4.61 | 62.38 ± 3.75 |
| CO_2_ (ppm) | 343.90 ± 10.51 | 342.85 ± 9.34 |

**Appendix Table 2** Results of correlation analysis of biomass (under both well-watered and moderate drought treatment), biomass difference and trait plasticity for 10 Poaceae species.

|  | RGR | R:S | Height | SLA | LA | RLWC | Photosynthetic rate | Transpiration rate |
| --- | --- | --- | --- | --- | --- | --- | --- | --- |
| Biomass (well-watered) | 0.485 | 0.247 | 0.358 | 0.221 | - 0.135 | - 0.459 | 0.365 | 0.488 |
| Biomass (moderate-drought) | 0.512 | 0.164 | 0.490 | - 0.201 | - 0.267 | - 0.250 | 0.108 | 0.324 |
| Biomass difference | 0.056 | 0.124 | 0.105 | 0.584 | 0.098 | 0.043 | 0.306 | 0.158 |

**Appendix Table 3** Mean (± SE) of the plasticity index (PI) of the species across the traits. Species are sorted from high to low PI. See Table 1 for the abbreviations of species.

| Species | Plasticity indices (PI) |
| --- | --- |
| BS | 0.119**±**0.029 |
| PP | 0.116**±**0.042 |
| PD | 0.107**±**0.041 |
| FO | 0.107**±**0.027 |
| PPr | 0.104**±**0.046 |
| AP | 0.090**±**0.038 |
| AG | 0.088**±**0.041 |
| SA | 0.071**±**0.024 |
| EN | 0.066**±**0.032 |
| DC | 0.046**±**0.019 |
